# Virtual High-Throughput Screening Identifies Mycophenolic Acid as a Novel RNA Capping Inhibitor

**DOI:** 10.1371/journal.pone.0024806

**Published:** 2011-09-15

**Authors:** Maude Tremblay-Létourneau, Simon Despins, Isabelle Bougie, Martin Bisaillon

**Affiliations:** RNA Group, Département de Biochimie, Faculté de Médecine et des Sciences de la Santé, Université de Sherbrooke, Sherbrooke, Québec, Canada; University of Cambridge, United Kingdom

## Abstract

The RNA guanylyltransferase (GTase) is involved in the synthesis of the ^m7^Gppp-RNA cap structure found at the 5′ end of eukaryotic mRNAs. GTases are members of the covalent nucleotidyl transferase superfamily, which also includes DNA and RNA ligases. GTases catalyze a two-step reaction in which they initially utilize GTP as a substrate to form a covalent enzyme-GMP intermediate. The GMP moiety is then transferred to the diphosphate end of the RNA transcript in the second step of the reaction to form the Gppp-RNA structure. In the current study, we used a combination of virtual database screening, homology modeling, and biochemical assays to search for novel GTase inhibitors. Using this approach, we demonstrate that mycophenolic acid (MPA) can inhibit the GTase reaction by preventing the catalytic transfer of the GMP moiety onto an acceptor RNA. As such, MPA represents a novel type of inhibitor against RNA guanylyltransferases that inhibits the second step of the catalytic reaction. Moreover, we show that the addition of MPA to *S. cerevisiae* cells leads to a reduction of capped mRNAs. Finally, biochemical assays also demonstrate that MPA can inhibit DNA ligases through inhibition of the second step of the reaction. The biological implications of these findings for the MPA-mediated inhibition of members of the covalent nucleotidyl superfamily are discussed.

## Introduction

The synthesis and maturation of eukaryotic mRNAs are crucial events for gene expression. During mRNA synthesis, eukaryotic mRNAs undergo a series of essential modifications before being exported to the cytoplasm where they are translated into proteins [1]. These processing events include the addition of a cap structure at the 5′ terminus, the splicing out of introns, the editing of specific nucleotides, and the acquisition of a poly(A) tail at the 3′ terminus. The RNA cap structure found at the 5′ end of mRNAs is critical for the splicing of the cap-proximal intron, the transport of mRNAs from the nucleus to the cytoplasm, and for both the stability and translation of mRNAs [2], [3]. The cap is synthesized by a series of three enzymatic reactions [4]. The first step involves the hydrolysis of the RNA 5′-triphosphate end of the nascent RNA by an RNA triphosphatase to form a diphosphate end. An RNA guanylyltransferase then catalyzes a two-step reaction in which it initially utilizes GTP as a substrate to form a covalent enzyme-GMP intermediate. The GMP moiety is then transferred to the diphosphate end of the RNA transcript in the second step of the reaction to form the GpppN structure. The guanosine residue is finally methylated by an RNA (guanine-N7)-methyltransferase to form the typical ^m7^GpppN cap structure.

A number of different microbial pathogens code for their own enzymes involved in the synthesis of a cap structure [5], [6], [7], [8], [9], [10]. Although the RNA cap structures originating from human and microbial enzymes are often identical, the physical organization of the genes, subunit composition, structure and catalytic mechanisms of the microbial-encoded enzymes involved in the synthesis of the RNA cap structure are often significantly different from those of host cells [2]. As a consequence these pathogenic cap-forming enzymes are potential targets for anti-microbial drugs.

**Figure 1:**
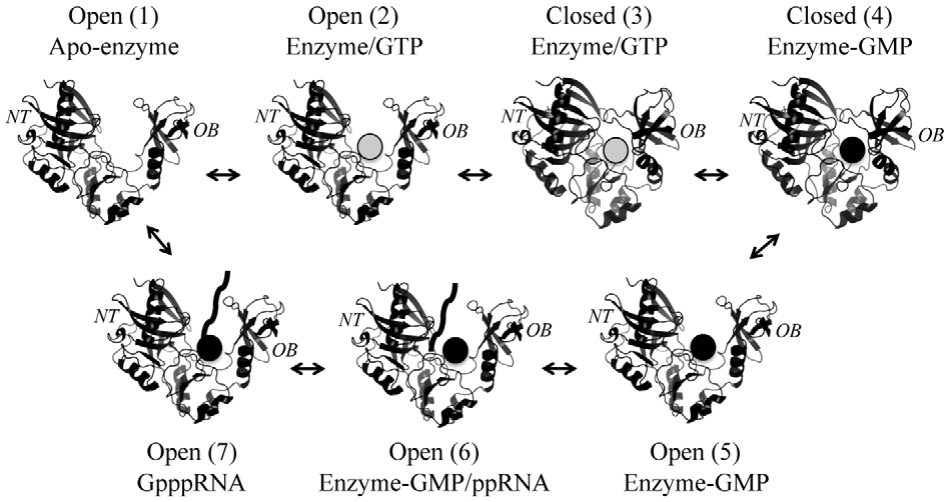
Structural and mechanistic pathway used by RNA guanylyltransferases. The mechanism for phosphoryltransfer involves conformational changes between an open and closed form of the enzyme [22], [23]. GTP (grey sphere) initially binds to the apo-enzyme (structure 1) which promotes closure of the N-terminal nucleotidyl transferase (NT) domain and the C-terminal oligomer-binding (OB) fold domain (structure 3). This is followed by hydrolysis of the GTP substrate to produce the enzyme-GMP covalent intermediate (structure 4). Hydrolysis of GTP disrupts the interactions between the bound guanylate and the C-terminal OB fold domain, thus destabilizing the closed form of the enzyme, which opens up with the concomitant release of pyrophosphate (structure 5). This exposes the RNA-binding site of the enzyme (exact location unknown), thereby allowing the subsequent transfer of the GMP moiety onto the acceptor RNA (structure 7). The capped RNA is then released and the enzyme can reinitiate the pathway.

During the past few years, both the RNA triphosphatase and the RNA (guanine-N7) methyltransferase (N7-MTase) components of the RNA capping machinery have been major targets for the development of drugs directed against RNA cap synthesis [11], [12], [13], [14], [15], [16], [17], [18], [19], [20]. Of all the enzymes involved in RNA capping, the RNA guanylyltransferase (GTase) has traditionally been considered a poor candidate as an anti-microbial target because of the high mechanistic and structural conservation of this enzyme across species [21]. Based on various crystal structures of GTases, a general mechanism for phosphoryltransfer has previously been elucidated which involves conformational changes between an open and closed form of the enzyme [22], [23]. In the first step of the reaction, GTP binds to the open form of the enzyme which promotes closure of the N-terminal nucleotidyl transferase (NT) domain and the C-terminal oligomer-binding (OB) fold domain. This closure is stabilized by interactions between the residues of the NT domain, the bound nucleotide, and residues on the OB fold domain. Domain closure is then followed by hydrolysis of the GTP substrate to produce the enzyme-GMP covalent intermediate. Hydrolysis of GTP disrupts the interactions between the bound guanylate and the C-terminal OB fold domain, thus destabilizing the closed form of the enzyme, which opens up with the concomitant release of pyrophosphate. This exposes the RNA-binding site of the enzyme, thereby allowing the subsequent transfer of the GMP moiety onto the acceptor RNA. Figure 1 summarizes the mechanistic and structural pathway used by GTases.

Recent *in vitro* studies have shown that foscarnet is a potent inhibitor of the GTase reaction [24]. Its mechanism of action is purported to occur through substrate binding inhibition on account of its analogous nature to pyrophosphate (PPi), a product of the GTase reaction. Ribavirin, a broad-spectrum nucleoside analogue approved for the treatment of various viral infections, is another inhibitor of the GTase activity [25]. Biochemical studies have shown that ribavirin triphosphate can actually be used as a substrate by the vaccinia virus GTase to form a covalent enzyme-ribavirin monophosphate intermediate reminiscent of the covalent enzyme-GMP intermediate [25]. Furthermore, ribavirin monophosphate can be transferred to the diphosphate end of an RNA transcript to form the unusual RpppN structure [25]. However, RNA transcripts blocked with ribavirin are not recognized efficiently by the cap-binding protein eIF4E, and are not translated into proteins [26]. The use of guanine-N7 methylation-inert cap donor molecules could potentially prove to be an interesting line of research for the development of anti-microbial drugs. However, on account of the possibility of off-targets, the risk of major side effects upon tratment with GTase substrate/product analogs remains. Several issues related to the specificity problem faced with these inhibitors can likely be partially resolved by the development of non-nucleoside inhibitors.

In the current study, we used a combination of virtual database screening, homology modeling, and biochemical assays to search for novel GTase inhibitors. We demonstrate that mycophenolic acid, a compound which is currently used both in cancer and immunosuppressive chemotherapy, is an inhibitor of the GTase reaction. The biological implications of these findings for the MPA-mediated inhibition of RNA capping are discussed.

## Materials and Methods

### Structure-based virtual screening

To identify potential candidate compounds that can bind to GTases, the crystal structures of various GTases were retrieved from the Protein Data Bank (*Chlorella* virus: 1CKN and 1CKO; *Candida albicans*: 1P16; *S. cerevisiae*: 3KYH). The chemical databases used in our virtual screening included the Sigma-Aldrich, Ambinter, ASINEX, IS Chemical technology, MolPort, and Vitas M Laboratory catalogs. Collectively, these 6 databases offered a collection of 13,800,000 small-molecule compounds.

The molecular docking program DOCK (Version 4.0) was used to perform the virtual screening [27]. Componds displaying at least 80% of structural similarity to GTP or ATP (>25,000 compounds) were screened for the binding to the four GTases. The levels of similarities were measured using the Tanimoto equation [28] and the PubChem dictionary-based binary fingerprint (http://pubchem.ncbi.nlm.nih.gov/). Based on the binding models of these compounds predicted by DOCK, the X-SCORE program (Version 1.1) was applied to obtain an estimate of the binding affinities of these compounds [29]. The compounds were then ranked according to their binding affinities as estimated by X-SCORE.

### Expression and purification of proteins

The RNA guanylyltransferases from *S. cerevisiae* (Ceg1), vaccinia virus (D1R), *Chlorella* virus (A103R), and human (HCE) were expressed and purified as described before [24], [25], [30], [31]. The ATP-dependent ligase DNA ligase from *Chlorella* virus (ChVLig) was also expressed and purified as described previously [32].

### Assay for enzyme-GMP complex formation

The assay was performed by incubating the enzyme (0.1 µM) with 10 µM [α-^32^P]GTP in a buffer containing 50 mM Tris-HCl, pH 8, 5 mM DTT, and 5 mM MgCl_2_ for 5 min at 30°C. The reactions were stopped by the addition of EDTA to 10 mM and SDS to 1%. The reactions were analyzed by electrophoresis through a 12.5% polyacrylamide gel containing 0.1% SDS. The radiolabeled proteins were visualized by autoradiography of the gel. The extent of covalent complex formation was quantitated by scanning the gel with a PhosphorImager (Amersham Biosciences).

### Preparation of RNA substrates

An RNA substrate of 81 nucleotides was synthesized with the MAXIscript kit (Ambion) using T7 RNA polymerase. The RNA transcript was synthesized from the pBS-KSII+ plasmid (Stratagene) that had been linearized with HindIII. The RNA substrate was purified on a denaturing 20% polyacrylamide gel and visualized by ultraviolet shadowing. The corresponding band was excised and then eluted from the gel by an overnight incubation in 0.1% SDS and 0.5 M ammonium acetate. The RNA was then precipitated with ethanol and quantitated by spectrophotometry. Alternatively, radiolabeled RNA substrates were also synthesized by adding [α-^32^P]ATP or [α-^32^P]GTP to the transcription reaction.

The purified 5′-triphosphorylated RNA was further processed to obtain a diphosphorylated 5′ end using the *S. cerevisiae* RNA 5′-triphosphatase (Cet1) which was expressed and purified as described before [33]. The diphosphorylated RNA (ppRNA) was precipitated with ethanol, resuspended, quantitated by spectrophotometry, and stored at −20°C.

### Molecular docking

Docking calculations were carried out using the Docking Server software and the Dreiding force field was used for energy minimization of MPA using built-in Chemaxon tools in Docking Server [34]. PM6 semi-empirical charges calculated by MOPAC2007 were added to the ligand atoms. Non-polar hydrogen atoms were merged and rotatable bonds were defined [35]. Docking calculations were carried out using the *Chlorella* virus and *Candida albicans* RNA guanylyltransferase crystal structures (Protein Data Bank 1CKN and 1P16). Essential hydrogen atoms, Kollman united atom type charges and solvation parameters were added with the aid of AutoDock tools [36]. Affinity (grid) maps of 20×20×20 Å grid points and 0.375 Å spacing were generated using the Autogrid program [36]. AutoDock parameter set- and distance-dependent dielectric functions were used in the calculation of the van der Waals and the electrostatic terms, respectively. Docking simulations were performed using the Lamarckian genetic algorithm and the Solis and Wets local search method [37]. Initial position, orientation, and torsions of the ligand molecules were set randomly. Each docking experiment was derived from two different runs that were set to terminate after a maximum of 2,500,000 energy evaluations. The population size was set to 150. During the search, a translational step of 0.2 Å, and quaternion and torsion steps of 5 Å were applied.

### UV-crosslinking experiments

UV-crosslinking between the internally ^32^P-labeled RNA of 81 nt and the yeast GTase was performed in a crosslink buffer (50 mM Tris pH 7.5, 5 mM DTT, 5 mM MgCl_2_). The protein (12 µM) was incubated with GTP (1 mM) and different concentrations of MPA for 10 minutes at 30°C. The radiolabeled RNA (3 µM) was added to the reaction mixture and incubated for 5 minutes at 30°C. The reaction mixture was exposed to UV light (254 nm, 20 Joules/cm^2^) for 5 min at 30°C using a Stratalinker 2400 UV Crosslinker (Stratagene). The crosslink mixture was denatured (50 mM Tris-HCl, pH 7.0, 5% sucrose, 5% b-mercaptoethanol, 2% SDS) and separated by electrophoresis on a 12% SDS-PAGE. The gel was analyzed by phosphorimaging.

### Ligation assay

The ligation reaction was performed as described previously [32]. Briefly, a 36 bp DNA duplex harboring a centrally placed nick was used as a substrate. The 18-mer constituting the 5′-phosphate-terminated strand 5′-d(ATTCCGATAGTGACTACA)-3′ was 5′-radiolabeled and gel purified as described before. This labeled 18-mer was then annealed to a complementary 36-mer in the presence of a 3′-OH 18-mer strand 5′-d(CATATCCGTGTCGCCCTT)-3′ [38], [39]. Ligation reaction mixtures containing 50 mM Tris-HCl (pH 7.5), 5 mM DTT, 10 mM MgCl_2_, 1 mM ATP, nicked duplex substrate and the *Chlorella* virus DNA ligase were incubated at 22°C for 15 min. The reactions were stopped by the addition of 1 µl 0.5 M EDTA and 5 µl formamide. The samples were heated at 95°C for 5 min and then analyzed by electrophoresis through a 17% polyacrylamide gel containing 7 M urea. The extent of ligation was determined by scanning the gel with a PhosphorImager (Amersham Biosciences).

### Primer extension analysis of 5′ ends

Primer extension reactions were performed as described previously [40] using a 5′ ^32^P-labeled 18-mer DNA oligonucleotide complementary to the 5′ region of the *SSA1* mRNA (positions +1 to +19). Total RNA was extracted form *S. cerevisiae* cells that were grown in the presence or absence of 500 µg/ml MPA for 3 h at 30°C. The primer extension reactions were analyzed by electrophoresis through a 8% polyacrylamide gel containing 7 M urea in TBE and visualized by autoradiography.

## Results and Discussion

### Identification of mycophenolic acid through virtual database screening

**Figure 2:**
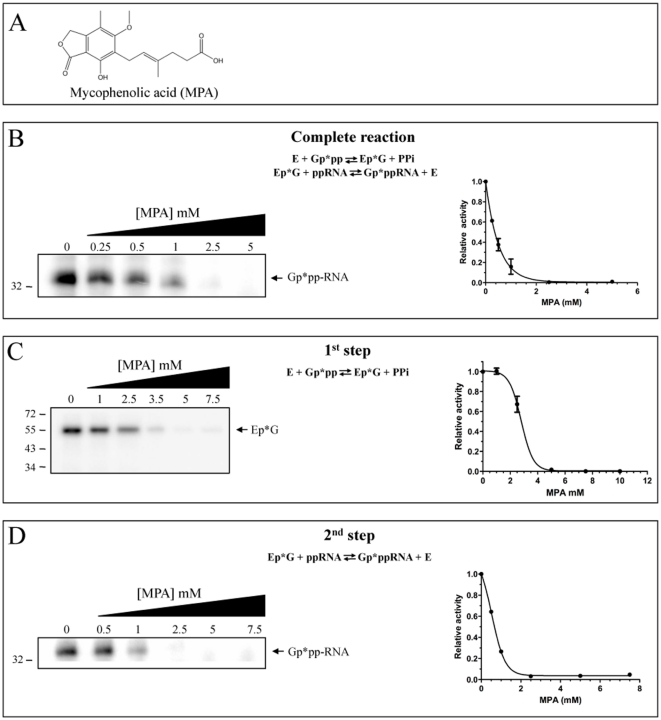
Mycophenolic acid inhibits the RNA guanylyltransferase activity. (**A**) Molecular structure of mycophenolic acid (MPA). (**B**) Increasing concentrations of MPA inhibit the complete RNA guanylyltransferase reaction. A standard GTase assay in which the purified enzyme (1 µM) was incubated with both [alpha-^32^P]GTP and a 5′-diphosphate acceptor RNA was performed in the presence of increasing concentrations of MPA. The reaction products were analyzed on a denaturing polyacrylamide gel and quantified (*right side of the panel*). (**C**) MPA is not a potent inhibitor of the first step of the GTase reaction. The formation of the enzyme-GMP covalent intermediate was monitored by incubating the purified enzyme (1 µM) in the presence of [alpha-^32^P]GTP and increasing concentrations of MPA. The radiolabeled covalent enzyme-GMP complex was then visualized by autoradiography following electrophoresis on a denaturing 12.5% polyacrylamide gel. The radiolabeled enzyme-GMP complex was quantified by phosphorimaging (*right side of the panel*). (**D**) The second step of the GTase reaction is inhibited by MPA. The transfer of the GMP moiety onto an acceptor RNA was evaluated by pre-incubating the enzyme (1 µM) with [alpha-^32^P]GTP (10 mM) to ensure formation of the radiolabeled covalent enzyme-GMP complex, followed by the addition of the acceptor 5′-diphosphate RNA (3 µM) in the presence of MPA. Formation of the radiolabeled capped GpppRNA was monitored following electrophoresis on a denaturant polyacrylamide gel. The radiolabeled GpppRNA was then quantified by phosphorimaging (*right side of the panel*).

We initially performed a virtual screen of more than 25,000 purine-related compounds for their ability to bind to the GTases of *Chlorella* virus (open and closed forms), *S. cerevisiae* (open form), and *C. albicans* (open form). The ligands were docked and ranked according to their respective docking scores. Our initial screen indicated that very few compounds bound to the open forms of these three different GTases. In all cases, the highest scoring compounds were not predicted to bind with very strong affinities to the enzymes (predicted *K*_d_>2 mM). However, one compound (mycophenolic acid) docked efficiently in the cavity of the closed form of the *Chlorella* virus GTase (predicted *K*_d_ of 280 µM). Mycophenolic acid (MPA, Fig. 2A) is a well-known inhibitor of inosine monophosphate dehydrogenase (IMPDH), a key cellular enzyme required for biosynthesis of guanine nucleotides [41]. Because T- and B-lymphocytes are critically dependent for their proliferation on *de novo* synthesis of purines, whereas other cell types can utilize salvage pathways, IMPDH inhibitors have potent cytostatic effects on lymphocytes [42], [43]. Accordingly, MPA has been used both in cancer and immunosuppressive chemotherapy as wll as in antiviral and antifungal therapy [44], [45], [46], [47], [48], [49], [50], [51], [52]. Because GTP is required for the transcription and replication of cellular and microbial genomes, it has traditionally been assumed that the decrease in the cytosolic concentration of GTP could affect both cell growth and the multiplication of fungal and viral pathogens.

### MPA inhibits the RNA guanylyltransferase activity

The ability of MPA to inhibit the GTase of the model organism *S. cerevisiae* (Ceg1 protein) was then investigated. The GTase activity is actually a two-step ping-pong reaction in which the enzyme first reacts with GTP to produce the enzyme–GMP (EpG) covalent intermediate with the concomitant release of pyrophosphate [2]. In the second step of the reaction, the GMP moiety is then transferred to a 5′-diphosphate RNA. The ability of MPA to inhibit the complete GTase reaction (both steps) was monitored using a standard GTase assay in which the purified enzyme was incubated with [alpha-^32^P]GTP and a 5′-diphosphate acceptor RNA. The reaction products were then analyzed on a denaturing polyacrylamide gel. As can be seen in figure 2B, the presence of increasing concentrations of MPA significanlty decreased the transfer of the radiolabeled GMP moiety onto the acceptor RNA. MPA was shown to inhibit the overall GTase activity by 50% at 360 µM, and by 80% at 900 µM.

In order to characterize the inhibition of the GTase activity by MPA, we next set out to investigate which of the two catalytic steps of the reaction is inhibited by MPA. The first step of the reaction, i.e. the formation of the enzyme-GMP covalent intermediate, was monitored by incubating the purified enzyme in the presence of [alpha-^32^P]GTP. The radiolabeled covalent enzyme-GMP complex was then visualized by autoradiography following electrophoresis on a denaturant polyacrylamide gel. Our results indicate that very high concentrations of MPA are required to inhibit the formation of the enzyme-GMP covalent complex (Fig. 2C). We determined that a concentration of 3 mM of MPA is required to inhibit the first step of the reaction by 50%, a concentration that corresponds to 8-times the amount required to inhibit the overall GTase reaction (both steps). The effect of MPA on the second step of the RNA reaction (i.e. the transfer of the GMP moiety onto an acceptor RNA) was next evaluated by pre-incubating the enzyme with GTP to ensure formation of the covalent enzyme-GMP complex, followed by the addition of the acceptor 5′-diphosphate RNA in the presence of MPA. Using such an approach, it was determined that a concentration of 640 µM of MPA is sufficient to inhibit 50% of the second step of the GTase reaction (Fig. 2D). Taken together, these results indicate that MPA inhibits the GTase reaction mainly through inhibition of the catalytic transfer of the GMP moiety onto an acceptor RNA.

### MPA is not a substrate for the RNA guanylyltransferase

**Figure 3:**
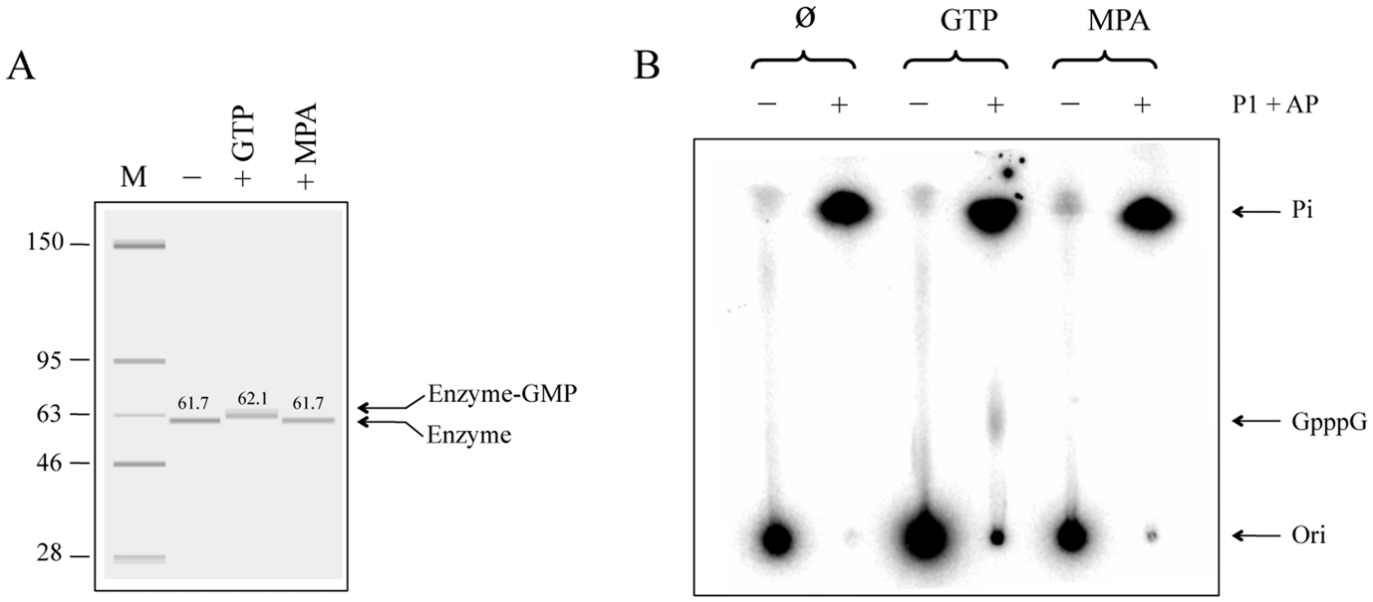
MPA is not a substrate for the RNA guanylyltransferase. (**A**) Capillary electrophoresis analysis of the RNA capping reaction. The GTase reactions were performed in the presence of the purified enzyme (1 µM) and GTP (1 mM) or MPA (1 mM), and the reaction products were analyzed by capillary electrophoresis. An untreated protein was also used as a control (-). The positions and sizes (in kDa) of the size markers (M) are indicated on the left. Masses are shown above the corresponding bands. (**B**) RNA capping reaction. The reaction mixtures contained 1 µg of purified enzyme, 23 pmol of radiolabelled 5′ diphosphate-terminated RNA (5′ p**p**G-RNA 3′, where the boldface indicates the radiolabelled moiety), and either 1 mM GTP or 1 mM MPA. An untreated control was also used in these assays (Ø). The reactions were incubated at 30°C for 30 min, and unincorporated nucleotides were removed by multiple rounds of ethanol precipitation. The RNAs were extracted with phenol/chloroform and recovered by ethanol precipitation. Aliquots of the RNA samples were adjusted to 50 mm NaOAc, pH 5.2, and digested with nuclease P1 (5 µg) for 60 min at 37°C. The reaction was then adjusted to 50 mm Tris-HCl, pH 8.0, and digested with alkaline phosphatase (1 unit) for 60 min at 37°C (*P1*+*AP*). The reaction products were analyzed by thin layer chromatography on a polyethyleneimine-cellulose plate developed with 0.5 M LiCl and 1 M formic acid. An autoradiogram of the plate is shown. The positions of the chromatographic origin (*ori*), inorganic phosphate (*Pi*), and GpppG are indicated.

In the typical GTase reaction, the nucleophilic attack on the α-phosphate of GTP by the enzyme during the first step of the reaction results in the formation of a covalent intermediate in which GMP is linked via a phosphoamide bond to a lysine residue of the enzyme [2]. Interestingly, it has previously been demonstrated that the nucleotide analog ribavirin triphosphate can be used as a substrate by the GTase to form an enzyme-RMP covalent intermediate [25]. Although MPA is not a nucleoside analog *per se*, we were still interested to monitor its ability to potentially be used as a substrate by the enzyme. The appearance of a slower migrating protein species is traditionally observed upon electrophoresis through a polyacrylamide gel when an RNA capping enzyme is incubated with GTP [25]. This slower migrating species corresponds to the enzyme with covalently bound GMP. We therefore incubated the enzyme in the presence of GTP or MPA and the polypeptide was analyzed by capillary electrophoresis. The appearance of a slower migrating protein species was observed repeatedly when the protein was incubated with GTP (Fig. 3A). However, this was not observed when the enzyme was incubated with MPA (Fig. 3A). We therefore conclude that MPA is not a substrate for the GTase. Accordingly, the transfer of MPA to an acceptor RNA could not be detected when the enzyme was incubated in the presence of MPA (Fig. 3B). This was tested by incubating the enzyme with an RNA substrate (81 nt) synthesized in the presence of [alpha-^32^P]GTP. This RNA substrate harbored a radiolabelled diphosphate 5′-end (5′ p**p**G-RNA 3′, where the boldface indicates the radiolabelled moiety) and 27 internally labelled guanosine residues. The RNA was then incubated with the GTase in the presence of GTP or MPA. The products of the reaction were extracted with phenol/chloroform and the RNA acceptor molecules were recovered by ethanol precipitation. Aliquots of the RNA samples were then digested with nuclease P1 and alkaline phosphatase and analyzed by polyethyleneimine-cellulose thin layer chromatography (Fig. 3B). The transfer of radiolabeled GMP to RNA was confirmed by demonstrating the release of a GpppG structure following digestion of the RNA samples with both nuclease P1 and alkaline phosphatase. However, the transfer of MPA to the acceptor RNA could not be detected when the enzyme was incubated in the presence of MPA (Fig. 3B). Overall, these results demonstrate that MPA is not a substrate for the GTase, and that it cannot be transferred to an acceptor RNA.

### Mechanistic/Structural implications

**Figure 4:**
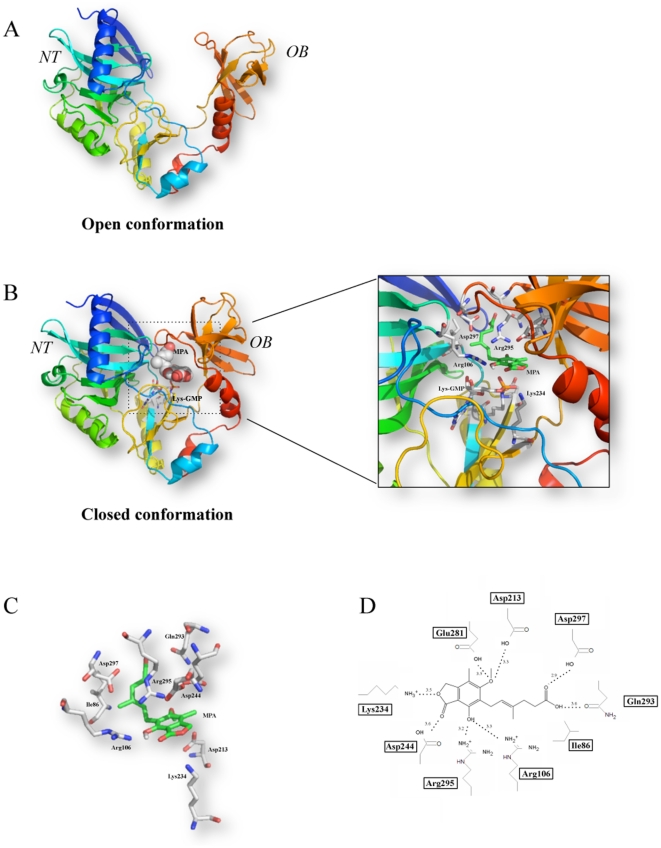
Molecular docking model for the binding of MPA to the closed form of the *Chlorella* virus GTase bound to GMP. (**A**) Ribbon diagram of the open form of the enzyme (PDB: 1CKO). The positions of the N-terminal nucleotidyl transferase (*NT*) and the C-terminal oligomer-binding (*OB*) fold domains are indicated. (**B**) Ribbon diagrams looking at the interaction of MPA with the GTase (PDB: 1CKN) bound to GMP. (**C**) Close-up view of the MPA binding pocket with emphasis on the residues interacting with MPA. (**D**) The side chains of of amino acids that are predicted to interact with MPA are shown, and the distances are indicated (*in Angströms*).

In order to better understand the mechanism by which MPA inhibits the transfer of GMP to RNA, we set out to use the power of molecular docking to provide information on the interaction between MPA and both the open and closed conformers of the RNA guanylyltransferase (Fig. 4A and B). Using extensive computational docking and structure optimizations, we generated models of MPA bound to the closed form enzyme–GMP complex of the *Chlorella* virus GTase. More than 2,000,000 energy evaluations were performed in order to provide an accurate description of the interactions. The models underwent 150 rounds of steepest descent energy minimization and did not contain energetically unfavorable bonds, angles or torsions.

Analysis of the molecular docking model predicts that MPA binds in the cleft created by the closure of the N-terminal and C-terminal domains. The space-filling model suggests that the molecular structure of MPA is sterically complementary to the cleft between the domains (Fig. 4B). The molecular docking model provides instructive findings on the interaction between specific residues of the enzyme and MPA (Fig. 4B). Arg106 and Asp213 of the N-terminal NT domain and Lys234, Asp244, Gln293, Arg295, Asp297, and Glu281 of the C-terminal OB fold domain would be involved in the coordination of the MPA through hydrogen-bonding (Fig. 4C). Ile86 (N-terminal) is predicted to make a hydrophobic interaction with MPA, which is located 4.0 Å away from the bound GMP (Fig. 4D).

**Figure 5:**
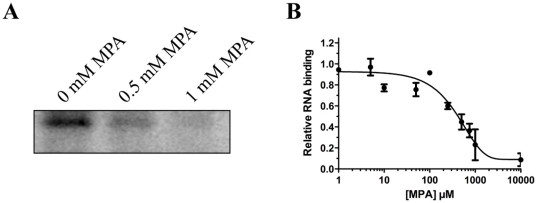
Binding of MPA to the enzyme-GMP complex inhibits the interaction with RNA. (**A**) The enzyme-GMP complex was incubated with a radiolabeled RNA substrate (3 µM) of 81 nucleotides in the presence of increasing concentrations of MPA. UV-cross-linking assays were performed to monitor the binding of radiolabelled RNA to the enzyme-GMP complex and visualized by SDS-PAGE analysis and autoradiography. (**B**) The reaction products were quantified by phosphorimaging.

As observed in our initial virtual screening, the molecular docking analyses suggest that MPA only binds to the closed form of the enzyme. No significant binding of MPA was detected when the molecular docking experiments were performed on the open forms of the *C. albicans*, *S. cerevisiae* and *Chlorella virus* RNA guanylyltransferases bound with GMP. It is tempting to speculate that the opening of the active site of the enzyme that is normally observed following the hydrolysis of GTP to produce the enzyme-GMP covalent intermediate [23] is inhibited by MPA. Following the formation of the GMP adduct, the enzyme must open up to provide access for the incoming mRNA substrate, since this site is blocked off in the closed form of the enzyme [23]. The presence of multiple interactions between MPA and residues of both the N-terminal and C-terminal domains of the enzyme likely inhibits this critical conformational change that is required for the binding of RNA. In order to validate this hypothesis, cross-linking assays were used to monitor the binding of radiolabelled RNA to the enzyme-GMP complex with or without MPA. An RNA harboring a 5′-triphosphate end was used as a ligand instead of the classical RNA with a 5′-diphosphate since the latter would lead to the transfer of the GMP moiety onto the RNA. Using this approach, an apparent *K*_d_ of 30 µM could be estimated for the binding of the RNA to the enzyme-GMP complex (data not shown). However, the binding of RNA to the enzyme-GMP/MPA complex was severely decreased in the presence of MPA (Fig. 5A). An IC_50_ value of 410 µM could be estimated from the inhibition assays (Fig. 5B). These results demonstrate that the presence of MPA inhibits the binding of the enzyme-GMP complex to RNA.

### Specificity

**Figure 6:**
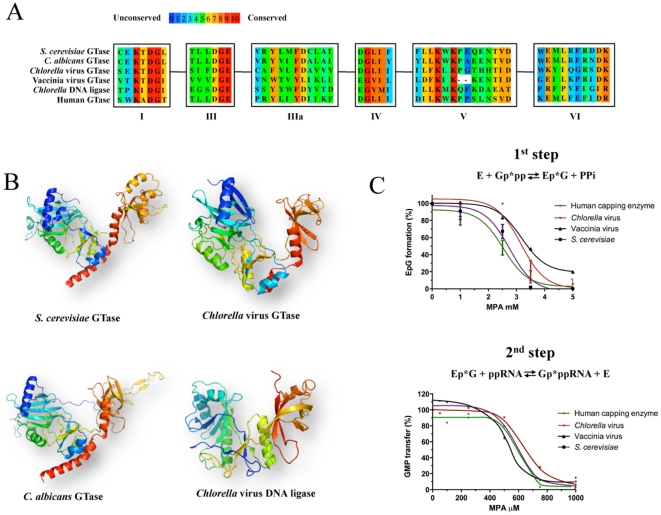
MPA inhibits the GTases of various origins. (**A**) Sequence conservation in members of the RNA/DNA nucleotidyltransferase superfamily. Members of this family share six conserved motifs (I, III, IIIa, IV, V, VI). An amino acid alignment of the GTases from *S. cerevisiae*, *C. albicans*, *Chlorella* virus, vaccinia virus, human, and the DNA ligase of *Chlorella* virus is presented. (B) Members of the RNA/DNA nucleotidyltransferase superfamily harbor a similar three-dimensional architecture consisting of an N-terminal NT domain and a C-terminal OB fold domain. The structures of the *S. cerevisiae* GTase (PDB: 3KYH), *C. albicans* GTase (PDB: 1P16), *Chlorella* virus GTase (PDB: 1CKN), and *Chlorella* virus DNA ligase (PDB: 1P8L) are shown. (C) The effect of MPA on the first and second step of the GTase reaction was monitored on the GTases from *S. cerevisiae*, *Chlorella* virus, vaccinia virus, and human. Both reactions were performed in the presence of increasing concentrations of MPA as described in the legend of figure 1.

GTases are members of the RNA/DNA nucleotidyltransferase superfamily that share six conserved sequence motifs (Fig. 6A) and a similar three-dimensional architecture consisting of an N-terminal NT domain and a C-terminal OB fold domain [21]. As observed previously, the NT domain (aa 1–243) of the *C. albicans* GTase aligns to the *Chlorella* virus GTase enzyme with 1.9 Å rmsd over 210 amino acids (26% side chain identity) [53]. In addition, the *C. albicans* GTase OB domain (aa 244–390) aligns to the *Chlorella* virus GTase OB domain with 1.8 Å rmsd over 72 amino acids (28% identity) [53]. Because of the high level of structural conservation between members of this family (Fig. 6B), we hypothesized that MPA should inhibit the GTases of various organisms. We therefore expressed and purified the RNA guanylyltransferases from vaccinia virus and *Chlorella* virus, as well as the corresponding human enzyme. Our results demonstrate that MPA inhibits all these GTases to the same extent than the *S. cerevisiae* homolog (Fig. 6C). IC_50_ values similar to the ones obtained for both the first (∼3 mM) and second step (∼620 µM) of the GTase reaction were observed when MPA was added to the reactions catalyzed by the vaccinia, *Chlorella*, and human enzymes. Overall, these results indicate that MPA inhibits the activity of different GTases through a similar mechanism that mainly prevents the catalytic transfer of the GMP moiety onto an acceptor RNA.

### Inhibition of DNA ligase

GTases are members of the covalent nucleotidyl transferase superfamily, which also includes DNA and RNA ligases [21]. The crystal structures of various family members revealed a common tertiary structure consisting of an N-terminal NT domain and a C-terminal OB-fold domain [22], [23], [53], [54], [55]. Based on both the sequence and structural similarities between GTases and DNA/RNA ligases (Fig. 6A and B), it is tempting to speculate that MPA might also be an inhibitor of DNA/RNA ligases. DNA/RNA ligation entails three sequential nucleotidyl transfer steps, and the first two steps are mechanistically related to the GTase reaction [56]. In the first step of the ligation reaction, nucleophilic attack on the α-phosphorus of ATP by ligase results in the formation of a covalent ligase-adenylate intermediate with the concomitant release of pyrophosphate. In the second step, AMP is transferred to the 5′-end of the 5′-phosphate-terminated DNA strand to form a DNA-adenylate complex. In the last step of the reaction, The the polynucleotides are joined with the concomitant release of AMP [56]. To verify the potential ability of MPA to inhibit DNA/RNA ligases, we have monitored the effect of MPA on the purified DNA ligase encoded by *Chlorella* virus.

**Figure 7:**
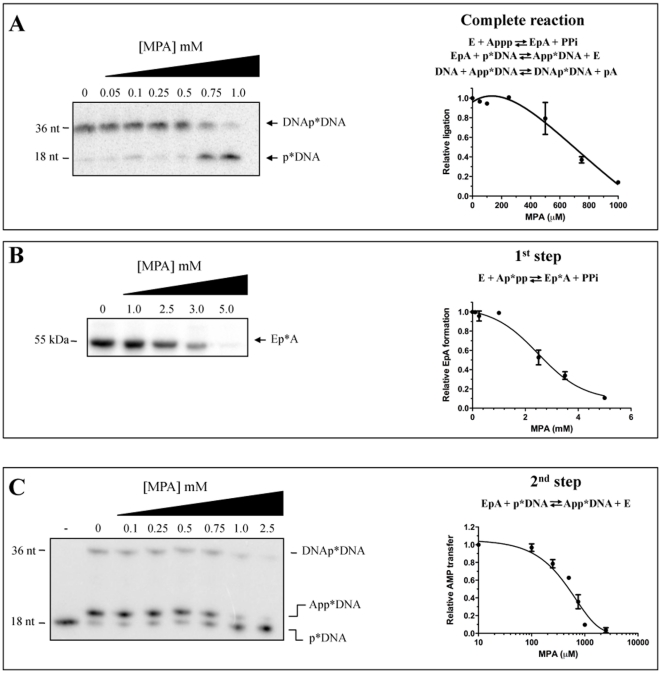
MPA inhibits the DNA ligase activity. (**A**) MPA inhibits the strand-joining activity of DNA ligase. Ligation reactions (50 mM Tris-HCl pH 7.5, 5 mM DTT, 10 mM MgCl_2_, 1 mM ATP, 500 fmol 1-nt gap DNA substrate and 6 pmol of the *Chlorella* virus DNA ligase) were performed at 22°C for 15 min in the presence of increasing concentrations of MPA. The samples were analyzed by electrophoresis through a 17% polyacrylamide gel containing 7 M urea. An autoradiogram of the gel is shown. The positions of the input 5′-monophosphate 18-mer strand (pDNA) and the 36-mer ligation product are indicated. The radiolabeled ligated product was then quantified by phosphorimaging (*right side of the panel*). (**B**) High concentrations of MPA are required to inhibit the first step of the ligase reaction. The formation of the enzyme-AMP covalent intermediate was monitored by incubating the purified enzyme in the presence of [alpha-^32^P]ATP and increasing concentrations of MPA. The radiolabeled covalent enzyme-AMP complex was then visualized by autoradiography following electrophoresis on a denaturant polyacrylamide gel. The radiolabeled enzyme-AMP complex was then quantified by phosphorimaging (right side of the panel). (**C**) The second step of the ligase reaction is inhibited by MPA. The transfer of the AMP moiety onto a radiolabeled 5′-monophosphate 18-mer strand (pDNA) was evaluated by pre-incubating the enzyme with ATP to ensure formation of the radiolabeled covalent enzyme-AMP complex, followed by the addition of a 1-nt gapped substrate in the presence of increasing concentrations of MPA. Conversion of the 5′-^32^P-labeled 18-mer strand into an adenylated species (AppDNA) was monitored by electrophoresis on a denaturant polyacrylamide gel. Lane 1: reaction performed in the absence of protein (-). The formation of the radiolabeled ApppDNA was quantified by phosphorimaging (*right side of the panel*).

The ability of MPA to inhibit the complete ligation reaction was monitored by incubating the purified enzyme with ATP and a DNA duplex containing a centrally located nick. The reaction products were then analyzed on a denaturing polyacrylamide gel. As can be seen in Fig. 7A, the presence of increasing concentrations of MPA significanlty decreased strand joining by the *Chlorella* virus DNA ligase. MPA was shown to inhibit the strand-joining activity by 50% at 700 µM. We next set out to investigate which of the first two catalytic steps of the ligation reaction is inhibited by MPA. The first step of the reaction, i.e. the formation of the enzyme-AMP covalent intermediate, was monitored by incubating the purified enzyme in the presence of [alpha-^32^P]ATP. The radiolabeled covalent enzyme-AMP complex was then visualized by autoradiography following electrophoresis on a denaturant polyacrylamide gel. Similarly to what was observed in the case of the GTase activity, our results indicate that very high concentrations of MPA are required to inhibit the first step of the reaction, i.e. the formation of the enzyme-AMP covalent complex (Fig. 7B). We determined that a concentration of ∼3.0 mM of MPA is required to inhibit the first step of the ligation reaction by 50%, a concentration that corresponds to 4-times the amount required to inhibit the overall reaction. The effect of MPA on the second step of the ligation reaction (i.e. the transfer of the AMP from ligase-adenylate to a 5′-phosphate terminus acceptor DNA) was next evaluated. In a typical ligation reaction, the adenylate-DNA intermediate would not be detected since the enzyme is highly efficient in ligating the two DNA strands [57]. However, as reported previously [58], the adenylate-DNA intermediate can accumulate to high levels when the enzyme acts on a substrate that contains a 1-nt gap between the reactive 3′-OH and 5′-phosphate strands. Using such an approach, the incubation of the enzyme with ATP and a gapped DNA substrate resulted in the conversion of the 5′-radiolabeled 18-mer strand into an adenylated species (AppDNA) that migrated 1-nt slower than the input 18-mer during polyacrylamide gel electrophoresis (Fig. 7C). The ability of MPA to inhibit this second step of the ligase reaction was investigated by pre-incubating the enzyme with ATP to ensure formation of the covalent enzyme-AMP complex, and by incubating the complex in the presence of the ligation substrate that contains a 1-nt gap between the reactive 3′-OH and 5′-phosphate strands. Using this strategy, it was determined that a concentration of 600 µM of MPA is sufficient to inhibit 50% of the second step of the ligation reaction (Fig. 7C). Taken together, these results indicate that MPA inhibits the ligation reaction mainly through inhibition of the second step of the reaction, i.e. the catalytic transfer of the AMP moiety onto the 5′-phosphate terminus of the nicked DNA substrate.

### Inhibition of RNA cap formation in cells

**Figure 8:**
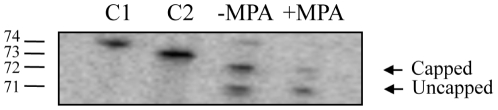
MPA inhibits RNA capping in *S. cerevisiae* cells. Primer extension assays were performed with a 5′ P^32^-labeled 18-mer oligonucleotide complementary to the 5′ region of the *SSA1* mRNA. The oligonucleotide was annealed to total mRNAs extracted from cells that were grown in the absence (*−MPA*) or presence (*+MPA*) of 500 µg/ml MPA for 3 h, and extended with reverse transcriptase. The primer extension reactions were analyzed by electrophoresis through a 8% polyacrylamide gel containing 7 M urea in TBE and visualized by autoradiography. Control P^32^-labeled RNA transcripts of 74 (*C1*) and 73 (*C2*) nucleotides were run in parallel. The positions and sizes (in nt) of the size markers are indicated on the *left*.

The effect of MPA on the capping of mRNAs was then assessed *in vivo*. A primer extension assay was used to monitor the effect of MPA on the formation of the RNA cap structure in *S. cerevisiae*. Previous studies have shown that during cDNA synthesis by reverse transcriptase, the presence of a cap structure results in the synthesis of products that harbor an extra 3′ nucleotide [40], [59]. We therefore performed primer extension assays with a 5′ P^32^-labeled 18-mer oligonucleotide complementary to the 5′ region of the *SSA1* mRNA. The oligonucleotide was annealed to total mRNAs extracted from cells that were grown in the presence or absence of 500 µg/ml MPA for 3 h, and extended with reverse transcriptase. Our results indicate that cDNA products with apparent chain lengths of 71 and 72 nucleotides, corresponding to uncapped and capped mRNAs, were synthesized using mRNAs extracted from untreated cells (Fig. 8). However, treatment with MPA resulted in a marked reduction of the 72 nucleotides species, indicating that cap formation was inhibited in the presence of MPA. Quantitative analysis indicate that cap formation was reduced by 50% in the presence of 500 µg/ml MPA (Fig. 8). We conclude that the addition of MPA to *S. cerevisiae* cells leads to an inhibiton of RNA cap synthesis.

### Conclusion

The current study provides the first biochemical evidence that MPA can directly interact with an RNA guanylyltransferase and inhibit its activity. We demonstrated that MPA inhibits the RNA guanylyltransferase reaction by preventing the catalytic transfer of the GMP moiety onto an acceptor RNA. Moreover, our RNA binding studies demonstrated that the binding of the enzyme-GMP intermediate to RNA is inhibited in the presence of MPA. As such, MPA represents a novel type of inhibitor against RNA guanylyltransferases that inhibits the second step of the catalytic reaction. Several inhibitors of the RNA guanylyltransferase activity have previously been identified [60]. However, these inhibitors all target the formation of the enzyme-GMP complex. For instance, foscarnet inhibits the formation of the enzyme-GMP intermediate on account of its analogous nature to pyrophosphate (PPi), a product of the RNA guanylyltransferase reaction [24]. Ribavirin, a broad-spectrum nucleoside analogue used as an antiviral for severe respiratory syncytial virus, Hepatitis C and other viral infections, is another example of an RNA guanylyltransferase inhibitor that prevents the formation of the enzyme-GMP intermediate [25]. Non-nucleoside competitive inhibitors have also been generated against the RNA guanylyltransferase of respiratory syncitial virus [61]. MPA, with its ability to inhibit the second step of the RNA guanylyltransferase reaction, has the potential to serve as a template for the development of more potent inhibitors. In fact, a number of MPA derivatives have been developed in recent years [62], [63]. Analysis of the interaction between these derivatives and RNA guanylyltransferases could shed light on the chemistry of the RNA capping reaction and lead to the development of more efficient anti-proliferative/anti-microbial drugs.

What is the biological relevance of the present finding? MPA has been shown to cause a reduction of the cellular GTP pools through the inhibition of IMPDH which is required for the *de novo* biosynthesis of GTP [41], [42]. A decrease in GTP concentrations could potentially have a negative effect on the capping of mRNAs. Evidence for this mechanism comes from studies performed with both the Sindbis virus and the Borna disease virus that showed that the viruses cannot replicate in cultured cells treated with ribavirin because the level of GTP falls too low to permit the capping of viral RNAs [48], [64], [65]. However, mounting evidence indicates that the antiproliferative/antimicrobial effect of MPA is not mediated entirely through the reduction of the intracellular GTP pool. For instance, MPA is not always a potent viral inhibitor [66]. The second mechanism by which MPA could inhibit the capping of mRNAs is by directly inhibiting the activity of GTases, as we demonstrated in the current study. Moreover, we do not exclude the possibility that MPA may have an increased frequency of utilization *in vivo* when the levels of GTP are lowered through the inhibition of IMPDH. In the present study, we demonstrated that MPA can inhibit RNA guanylyltransferases and ligases. MPA therefore appears as a pleiotropic agent that may function through multiple targets (IMPDH, RNA guanylyltransferase, ligases), as recently suggested through proteomic analysis [67]. As such, MPA shares many properties with ribavirin. Both compounds lead to a reduction of the *de novo* synthesis of GTP through IMPDH inhibition, and both molecules can inhibit RNA capping, albeit through different mechanisms of inhibition.
